# Body size and the risk of biliary tract cancer: a population-based study in China

**DOI:** 10.1038/sj.bjc.6604616

**Published:** 2008-08-26

**Authors:** A W Hsing, L C Sakoda, A Rashid, J Chen, M C Shen, T Q Han, B S Wang, Y T Gao

**Affiliations:** 1 Division of Cancer Epidemiology and Genetics, Department of Health and Human Services, National Cancer Institute, Bethesda, MD, USA; 2 Department of Epidemiology, University of Washington, Seattle, WA, USA; 3 Department of Pathology, MD Anderson Cancer Center, Houston, TX, USA; 4 Department of Epidemiology and Biostatistics, University of Pennsylvania, Philadelphia, PA, USA; 5 Shanghai Tumor Hospital, Fudan University, Shanghai, China; 6 Department of Surgery, Ruijin Hospital, Shanghai, China; 7 Zhongshan Hospital, Fudan University, Shanghai, China; 8 Shanghai Cancer Institute, Shanghai, China

**Keywords:** biliary tract cancer, gallstones, obesity, body mass index, waist-to-hip ratio, China

## Abstract

Though obesity is an established risk factor for gall bladder cancer, its role in cancers of the extrahepatic bile ducts and ampulla of Vater is less clear, as also is the role of abdominal obesity. In a population-based case–control study of biliary tract cancer in Shanghai, China, odds ratios (ORs) and 95% confidence intervals (CIs) were calculated for biliary tract cancer in relation to anthropometric measures, including body mass index (BMI) at various ages and waist-to-hip ratio (WHR), adjusting for age, sex, and education. The study included 627 patients with biliary tract cancer (368 gall bladder, 191 bile duct, 68 ampulla of Vater) and 959 healthy subjects randomly selected from the population. A higher BMI at all ages, including early adulthood (ages 20–29 years), and a greater WHR were associated with an increased risk of gall bladder cancer. A high usual adult BMI (⩾25) was associated with a 1.6-fold risk of gall bladder cancer (95% CI 1.2–2.1, *P* for trend <0.001). Among subjects without gallstones, BMI was also positively associated with gall bladder cancer risk. Regardless of BMI levels, increasing WHR was associated with an excess risk of gall bladder cancer risk, with those having a high BMI (⩾25) and a high WHR (>0.90) having the highest risk of gall bladder cancer (OR=12.6, 95% CI 4.8–33.2), relative to those with a low BMI and WHR. We found no clear risk patterns for cancers of the bile duct and ampulla of Vater. These results suggest that both overall and abdominal obesity, including obesity in early adulthood, are associated with an increased risk of gall bladder cancer. The increasing prevalence of obesity and cholesterol stones in Shanghai seems at least partly responsible for the rising incidence of gall bladder cancer in Shanghai.

Biliary tract cancers, which include cancers of the gall bladder, extrahepatic bile duct, and ampulla of Vater, are rare but highly fatal (Hsing *et al*, 2006). Apart from gallstones, the aetiology of biliary cancers is obscure (Wistuba and Gazdar, 2004; Hsing *et al*, 2007c). Obesity is closely linked to both gallstones and gall bladder cancer (Jorgensen, 1989; Kato *et al*, 1992; Stampfer *et al*, 1992; Hsing *et al*, 2006), although its relationship with other biliary cancers is less clear. It is also unclear at what age excess body fat most affects disease risk, as measurements of body size have been mainly related to a single point in time, and whether obesity independently influences biliary cancer risk, given the close connection between obesity and gallstone risk and between gallstones and biliary tract cancer. In addition, the role of abdominal obesity or body fat distribution in biliary tract cancer are unclear, although abdominal obesity is more closely associated with lipid and hormone metabolism (Giorgino *et al*, 2005).

Thus, to clarify further the role of overall and abdominal obesity in biliary tract cancer, in a large population-based case–control study in Shanghai, China, we have examined the roles of body mass index (BMI), both usual (adult) and at various time points in life, and waist-to-hip ratio (WHR).

## Materials and methods

The study protocol was approved by the Institutional Review Boards of the US National Cancer Institute (NCI) and the Shanghai Cancer Institute (SCI). Details of the study have been reported elsewhere (Hsing *et al*, 1998, 2007a, 2007b, 2007c, 2007d). Patients with primary biliary cancer (ICD-9, 156) newly diagnosed between June 1997 and May 2001 were identified through a rapid-reporting system established between the Shanghai Cancer Institute and 42 collaborating hospitals in urban Shanghai. Judging from incidence data reported to the Shanghai Cancer Registry, this system captured over 95% of all biliary cancer cases diagnosed within the study period. Both independent and consensus review of histologic data by expert pathologists positively confirmed the reported diagnoses for more than 70% of the cancer cases. The remaining cancer cases were confirmed through clinical review of medical imaging data from computed tomography, magnetic resonance imaging, ultrasonography, or endoscopic retrograde cholangiopancreatography. In addition, healthy individuals were randomly chosen as population controls, frequency-matched on the age and sex of the cancer case, using the demographic information recorded on the personal registration cards of the Shanghai Resident Registry.

In-person interviews were conducted using a structured questionnaire to elicit information on potential risk factors, including demographic characteristics, cigarette and alcohol use, medical history, diet, and family history of cancer. All interviews were tape-recorded and reviewed to verify that these data were recorded accurately and to ensure that each interview was conducted properly. In addition, 5% of the study subjects were randomly selected and re-interviewed, with a concordance rate of over 90% on responses to key questions across the two interviews. Of those eligible, 627 cancer patients and 959 population control subjects agreed to participate and provided written informed consent at enrollment, a response rate of over 90% for cases and 82% for controls. Among participating controls, 85% also consented to abdominal ultrasound screening for gallstone detection. Individuals were defined as having biliary stones if they had previously undergone cholecystectomy or had gallstones.

At the interview, subjects were asked for their adult height, current weight; usual weight 5 years before interview, usual weight during specific age decades (i.e., at ages 20–29, 30–39, 40–49, 50–59 years), maximum adult weight (excluding pregnancy weight, for women), age at and duration of maximum weight, main area of weight gain on the body, and history of adult weight cycling (⩾7 kg gain or loss). Physical measurements of standing height, weight, and waist and hip circumference were also taken at the interview. Each measure was taken two times. When the difference between the two measurements exceeded predetermined tolerance limits (height, 2 cm; weight, 1 kg; waist and hip circumference, 2 cm), an additional measurement was taken. Final measurements for height, weight, and waist and hip circumference were determined by averaging the two closest values.

### Statistical analysis

BMI, weight in kilograms divided by the square of height in meters (kg/m^2^), was used to assess overall obesity. Self-reported data were used to calculate BMI at specific age periods and maximum adult BMI, respectively. The change in BMI over time, defined as the difference in BMI between age intervals (e.g., from the ages 20–29 to 30–39 years), was also computed. Subjects were classified into the BMI categories established by the World Health Organization (WHO) for Asian populations, with the BMI category of 18.5–22.9 kg/m^2^, defined as ‘normal’ and used as the reference group (WHO, 2000). Body mass index categories of ‘overweight’ (23.0–24.9) and ‘obesity’ (⩾25) were combined when the number of cases in either category was small. For other anthropometric measures, such as height and BMI change, subjects were categorized according to either the tertile or quartile distributions of the controls. WHR, waist circumference divided by hip circumference, was used as a measure of abdominal adiposity. As bile duct and ampulla of Vater cases are diagnosed at a late stage, data on waist and hip circumference are less reliable, and WHR was not computed for these two subsites.

Unconditional logistic regression was used to calculate odds ratios and 95% confidence intervals for each cancer subsite associated with BMI and other anthropometric measures, adjusting for age, sex, and education. Gall bladder cancer patients were compared with controls without a history of cholecystectomy (*n*=49), whereas patients with bile duct or ampullary cancer were compared with all control subjects. Additional covariates, including cigarette smoking, alcohol use, and history of diabetes and hypertension, were evaluated as potential confounding variables but were not included in the final models, as they did not appreciably change the risk estimates. Sex-specific analyses were performed to evaluate differences between men and women in risk associated with body size. Tests for linear trend in risks of cancer were conducted when appropriate, with anthropometric variables evaluated on a continuous scale. All tests were two-sided, with *P*<0.05 defined as statistically significant.

## Results

Table 1 shows selected characteristics of study subjects. As shown, more women were diagnosed with gall bladder cancer than men, whereas slightly more men were diagnosed with cancers of the extrahepatic bile duct and ampulla of Vater. Cases and controls were similar in age. Relative to controls, patients with gall bladder cancer were less educated. Gall bladder cancer case patients were less likely to smoke cigarettes and drink alcohol regularly than control subjects, whereas smoking was more common among those with bile duct and ampullary cancers. Cancer patients were more likely to have gallstones and diabetes.

Table 2 shows the risk of biliary cancer by subsite in relation to body size. Quartile cutoffs were used for gall bladder cancer, whereas tertiles were used for extrahepatic bile duct and ampulla of Vater cancers because of their smaller numbers of cases. All BMI-related variables were positively associated with gall bladder cancer risk, after adjustment for age, gender, and education level. BMI in early adulthood (ages 20–29 years) remained significant after adjustment for gallstones (OR=2.04, 95% CI: 0.9–4.8, *P* for trend=0.01) or among subjects without gallstones (OR=3.7, 95% CI 1.1–12.1) (data not shown). Although larger waist circumference was only slightly associated with an increased risk of gall bladder cancer, a high WHR (>0.897) was associated with a 4.7-fold risk (95% CI 3.1–7.2, *P* for trend <0.0001). There was no clear association with cancers of the extrahepatic bile duct or ampulla of Vater. Timing and duration of maximum adult weight, primary location of weight gain, and weight cycling did not appear to be related to risk for stones or cancer (data not shown), although too few subjects reported a history of weight cycling for reliable assessment.

Table 3 presents gall bladder cancer risk cross-classified by four levels (quartiles) of BMI and three levels (tertiles) of WHR. Regardless of BMI similarly increasing levels of WHR were strongly associated with excess risk of gall bladder cancer, with WHR having a greater impact than BMI. The highest risk is seen for subjects with both a high BMI and a high WHR, with those in the highest categories of BMI (⩾25) and WHR (>0.87) having an 8.3-fold risk of gallstones (95% CI 4.6–14.9) and a 12.6-fold risk of gall bladder cancer (95% CI 4.8–33.2), relative to subjects with a low BMI and WHR.

## Discussion

In this population-based study, WHR and BMI at all ages, including early adulthood, were associated with an increased risk of gall bladder cancer. These results suggest that both overall and abdominal obesity play an important role in the aetiology of gall bladder cancer. It seems likely that the increasing prevalence of obesity and cholesterol stones in Shanghai is at least partly responsible for the rising incidence of gall bladder cancer in Shanghai. These findings for gall bladder cancer are consistent with those of previous studies (Zatonski *et al*, 1997; Wolk *et al*, 2001; Calle *et al*, 2003; Samanic *et al*, 2004; Engeland *et al*, 2005). We found that the effect of obesity on gall bladder cancer is not mediated entirely by gallstones, as the BMI effect persisted after adjustment for gallstones. Among those without gallstones, BMI is also associated with gall bladder cancer risk. However, it should be noted that the statistical power for analysis stratified by gallstones was limited, since less than 20% of the gall bladder cancer patients had no history of stones and less than 25% of the controls had a history of stones. Although gallstones are closely linked to both BMI and gall bladder cancer, in assessing the effect of BMI, it may not be necessary to adjust for gallstones, which are thought to be an important factor in the causal pathway. Thus, adjustment for gallstones would attenuate the true association between obesity and biliary cancer risk. However, the gallstone-adjusted results suggest that obesity may also increase the risk of gall bladder cancer through pathways not related to gallstone pathogenesis. In addition to affecting lipid metabolism, obesity can affect the risk of gallstones and gall bladder cancer through adverse changes in the metabolism of endogenous hormones, including sex steroids, sex hormone-binding globulin, insulin growth factor-I, and inflammatory mediators, such as insulin and cytokines; all of these stimulate cell proliferation and inhibit apoptosis, thereby enhancing the potential for tumour growth (Coussens and Werb, 2002).

Despite the fact that our study is the largest population-based study to date, we found no clear association between obesity and cancer of the bile duct or ampulla of Vater, possibly because of their small numbers. Of the five available studies assessing bile duct cancer risk specifically (Chow *et al*, 1994, 1999; Samanic *et al*, 2004; Oh *et al*, 2005), one reported an increased risk in white men but not white women, two small studies reported no association between obesity and ampullary cancer (Chow *et al*, 1999), whereas one large study showed elevated risk of ampullary cancer in US black men (Samanic *et al*, 2004). In two studies, tumours of the extrahepatic bile duct and/or the ampulla of Vater were combined with gall bladder cancer and were not evaluated separately (Lew and Garfinkel, 1979; Engeland *et al*, 2005). As molecular and epidemiologic studies suggest that these three subsites have different aetiologies, future studies should evaluate the role of obesity in extrahepatic bile duct and ampulla of Vater separately from gall bladder cancer.

In this report, we used the WHO-recommended BMI cutoffs for Asians, as they have been shown to have different body composition and bone density (Pan *et al*, 2004). It has also been shown that at a much lower level of BMI, relative to Caucasians, Asians have a high prevalence of diabetes, metabolic syndrome, and other adverse outcomes, suggesting that lower BMI cutoffs should be used for these relatively lean populations. Based on the WHO Asian-specific cutoffs, the prevalence of obesity (BMI⩾25 kg/m^2^) in the Shanghai population is 25% but it is only 4.1% if the conventional cutoffs (BMI >30 kg/m^2^) are used. It should be noted that although median BMI in our population is low (22.9 kg/m^2^), the prevalence of abdominal obesity (WHR>0.9) is quite high (50%).

The validity of our results hinges largely on the validity of self-reported BMI, which appeared to be quite good, given the fact that, among our population controls, self-reported current weight correlated well with measured weight (*r*=0.89, *P*<0.0001) and there is a good correlation between reported BMI and measured WHR, suggesting that misclassification by BMI is minimal. The validity of recalling weight for each decade in adulthood is likely to be lower than that of current weight. However, in our study correlations between reported weights in various decades of life were high (>0.8) and misclassification, if any, is likely to be non-differential in cases and controls.

Several strengths of the study should be noted. Selection bias was minimal because of population-based design with high case ascertainment and high participation rates. Misclassification of cases was minimal, given the nearly complete confirmation of case status, achieved by comprehensive pathology and clinical review. As the largest study to date, we were able to investigate the role of obesity by anatomic subsite.

One major limitation of our study is the potential disease effect on the reporting and measurement of body size. As cancer can cause wasting and weight loss, cases might be expected to have lower body weight than controls. However, we found that cases actually had a higher BMI than controls, suggesting that the weight, especially usual adult weight, in our cases had not been greatly affected by cancer. We cannot generalize our results to western populations, because of the large differences in prevalence of obesity and the fact that we were unable to evaluate the effect of BMIs greater than 30, as only 4% of the controls had a BMI that large.

In summary, this population-based study in Shanghai confirmed that overall obesity is an important risk factor for gall bladder cancer and found that obesity in early adulthood and abdominal obesity are also relevant. Given the epidemic of obesity worldwide and the substantial burden of gallstones in most populations, a strategy to slow down the rising obesity trend and to minimize the burden of gallstones and biliary cancer would be important to develop.

## Figures and Tables

**Table 1:** Selected characteristics of study subjects by case–control status, Shanghai, China

	**Cancer cases**			**Population controls**
	**Gall bladder**	**Bile duct**	**ampulla of Vater**	**All**
	***N* (%)**	***N* (%)**	***N* (%)**	***N* (%)**
Total	368 (100.0)	191 (100.0)	68 (100.0)	959 (100.0)
				
*Sex*				
Male	99 (26.9)^a^	99 (51.8)^b^	37 (54.4)^b^	373 (38.9)
Female	269 (73.1)	92 (48.2)	31 (45.6)	586 (61.1)
				
*Age at interview*				
34–44	19 (5.1)	6 (3.1)	1 (1.5)	41 (4.3)
45–54	30 (8.2)	24 (12.6)	5 (7.4)	87 (9.1)
55–64	96 (26.1)	47 (24.6)	20 (29.4)	269 (28.0)
65–74	223 (60.6)	114 (59.7)	42 (61.8)	562 (58.6)
Mean (SD)	64.2 (8.5)	63.6 (8.4)	65.2 (7.1)	63.9 (8.4)
				
*Education*				
None/primary	198 (53.8)^a^	86 (45.0)	29 (42.7)	396 (41.3)
Junior middle	79 (21.5)	43 (22.5)	16 (23.5)	233 (24.3)
Senior middle	50 (13.6)	31 (16.2)	15 (22.1)	190 (19.8)
Some college	41 (11.1)	30 (15.7)	8 (11.8)	140 (14.6)
Cigarette use^c^	89 (24.2)^a^	76 (39.8)^b^	30 (44.1)^b^	285 (29.7)
Alcohol consumption^d^	52 (14.1)^a^	50 (26.2)	15 (22.1)	198 (20.7)
History of hypertension	138 (37.5)	61 (31.9)^b^	20 (29.4)^b^	406 (42.3)
History of diabetes	51 (13.9)^a^	20 (10.5)	5 (7.4)	78 (8.1)
Biliary stones	308 (83.7)^a^	127 (66.5)^b^	36 (52.9)^b^	224 (23.4)

a Compared with population controls with no prior cholecystectomy, *P*<0.05.

b Compared with all population controls, *P*<0.05.

c History of at least one cigarette per day for 6 or more months.

d Consumption of alcohol at least once a week for 6 or more months.

**Table 2:** Odds ratios (ORs) and 95% confidence intervals (CIs) for biliary tract cancer in relation to anthropometric measures

		**Gall bladder cancer (*N*=368)**				**Extrahepatic bile duct cancer (*N*=191)**				**ampulla of Vater cancer (*N*=68)**		
**Anthropometry (Quartile)^a^**	**N1/N2^b^**	**OR^c^**	**95 % CI**	***P*_trend_**	**Anthropometry^a^ (Tertile)**	**N3/N4/N5^d^**	**OR^c^**	**95 % CI**	***P*_trend_**	**OR^c^**	**95% CI**	***P*_trend_**
Height (cm)					Reported height (cm)							
⩽156	243/114	1.0	—		⩽158							
156–162	254/117	1.11	0.81–1.52		159–165	355/56/24	1	—		1	—	
163–168	208/68	1.12	0.75–1.68		>165	310/65/16	1.08	0.70–1.67		0.58	0.27–1.23	
>168	197/67	1.8	1.03–3.16	*0.11*		294/68/28	0.79	0.44–1.42	*0.47*	0.65	0.25–1.67	*0.36*
												
Usual adult BMI (kg/m^2^)					Usual adult BMI (kg/m^2^)							
<18.5	78/17	0.62	0.35–1.09									
18.5–22.9	390/130	1	—		<18.5	79/8/1	0.52	0.24–1.13		—	—	
23.0–24.9	184/73	1.2	0.85–1.68		18.5–22.9	404/86/29	1	—		1	—	
⩾25.0	249/145	1.56	1.17–2.10	<*0.001*	⩾23.0	475/95/38	0.99	0.71–1.37	*0.29*	1.16	0.70–1.93	*0.1*
												
Maximum adult BMI (kg/m^2^)					Maximum adult BMI							
<18.5	17/6	1.24	0.47–3.29									
18.5–22.9	259/74	1	—		<18.5	17/2/0	0.58	0.13–2.63		—	—	
23.0–24.9	212/83	1.35	0.94–1.95		18.5–22.9	266/55/21	1	—		1	—	
⩾25.0	397/185	1.48	1.08–2.03	*0.02*	⩾23.0	659/123/44	0.91	0.64–1.30	*0.84*	0.89	0.52–1.54	*0.94*
												
*BMI over time (kg/m*^*2*^)												
Ages 20–29 (years)					Ages 20–29 (years)							
<18.5	227/61	0.73	0.53–1.02									
18.5–22.9	533/199	1	—		<18.5	240/25/5	0.52	0.33–0.82		0.26	0.10–0.66	
23.0–24.9	19/16	1.46	0.94–2.25		18.5–22.9	563/119/47	1	—		1	—	
⩾25.0	65/39	2.03	1.02–4.06	<*0.001*	⩾23.0	95/20/13	1.07	0.63–1.82	*0.01*	1.02	0.44–2.35	*0.01*
Ages 30–39 (years)					Ages 30–39 (years)							
<18.5	124/36	0.95	0.63–1.43									
18.5–22.9	526/162	1	—		<18.5	132/16/2	0.64	0.36–1.12		0.2	0.05–0.86	
23.0–24.9	126/77	1.9	1.35–2.66		18.5–22.9	557/112/45	1	—		1	—	
⩾25.0	60/42	1.97	1.27–3.06	<*0.001*	⩾23.0	200/31/13	0.82	0.53–1.27	*0.67*	0.91	0.47–1.74	*0.13*
Ages 40–49 (years)					Ages 40–49 (years)							
<18.5	74/18	0.74	0.43–1.29									
18.5–22.9	443/144	1	—		<18.5	77/8/0	0.57	0.26–1.22		—	—	
23.0–24.9	150/68	1.37	0.97–1.94		18.5–22.9	465/89/35	1	—		1	—	
⩾25.0	126/72	1.6	1.13–2.27	*0.001*	⩾23.0	302/49/19	0.89	0.60–1.30	*0.68*	0.9	0.50–1.62	*0.24*
Ages 50–59 (years)					Ages 50–59 (years)							
<18.5	49/11	0.65	0.32–1.32									
18.5–22.9	312/100		—		<18.5	50/6/1	0.69	0.28–1.70		0.3	0.04–2.27	
23.0–24.9	1	1.19	0.80–1.75		18.5–22.9	329/62/25	1	—		1	—	
⩾25.0	102/156	1.88	1.33–2.64	<*0.001*	⩾23.0	328/59/24	1	0.67–1.48	*0.62*	1.01	0.56–1.82	*0.44*
BMI change in adulthood^d^					BMI change in adulthood^c^							
⩽0.74	211/74	1	—		⩽1.66	298/64/22	1	—		1	—	
0.75–2.77	211/62	0.93	0.62–1.39		1.67–4.20	295/51/17	0.86	0.57–1.30		0.81	0.42–1.57	
2.78–5.21	211/86	1.45	0.98–2.14	*0.01*	>4.20	305/49/20	0.88	0.58–1.34	*0.54*	1.09	0.57–2.09	*0.82*
>5.21	211/93	1.47	1.00–2.16									
Waist to hip Ratio^e^												
⩽0.81	228/38	1	—									
0.81–0.852	228/51	1.38	0.87–2.20									
0.853–0.897	223/90	2.71	1.76–4.18									
>0.897	223/133	4.72	3.08–7.23	<*0.0001*								

a For categories of height and BMI change, quartile and tertile cutoff points (quartile or tertile) were based on their distribution among population controls without cholecystectomy.

b N1 controls without a history of cholecystectomy, N2 gall bladder cancer cases, N3 controls, N4 extrahepatic bile duct cancer cases, N5 ampulla of Vater cases.

c Adjusted for age (continuous), sex (male, female), and education (none/primary, junior middle, senior, and some college).

d Change between usual adult BMI and BMI at ages 20–29 years; Also adjusted for BMI at ages 20–29 years.

e WHR was not computed for extrahepatic bile duct or ampulla of Vater cancers.

**Table 3:** Odds ratios (ORs)^a^ and 95% confidence intervals (CIs) for gall bladder cancer in relation to BMI and WHR

	**BMI^b^**							
	**<18.5**		**18.5–22.9**		**23.0–24.9**		**⩾25**	
**WHR^c^**	**N_1_/N_2_^d^**	**OR (95% CI)**	**N_1_/N_2_**	**OR (95% CI)**	**N_1_/N_2_**	**OR (95% CI)**	**N_1_/N_2_**	**OR (95% CI)**
<0.82	(56/5)	1.0 (—)	(144/27)	2.22 (0.81–6.11)	(49/67)	1.65 (0.48–5.58)	(56/6)	1.01 (0.29–3.56)
0.82–0.87	(12/4)	4.25 (0.97–18.6)	(130/38)	3.87 (1.43–10.4)	(59/21)	4.19 (1.46–11.9)	(96/29)	3.70 (1.15–8.74)
>0.87	(10/3)	4.37 (0.87–21.8)	(114/44)	5.70 (2.1–15.4)	(76/36)	7.54 (2.73–20.3)	(97/92)	12.6 (4.77–33.2)

a Adjusted for age (continuous), sex (male, female), and education (none/primary, junior middle, senior, and some college).

b Usual adult BMI; Based on WHO cutoffs.

c Waist-to-hip ratio: based on distribution among controls.

d N1 controls. N2 gall bladder cancer cases.
